# Effects of integrin-linked kinase on protein kinase b, glycogen synthase kinase-3β, and β-catenin molecules in ovarian cancer cells 

**DOI:** 10.22038/IJBMS.2021.58716.13042

**Published:** 2021-11

**Authors:** Seda Mehtap Sarı Kılıçaslan, Zerrin İncesu

**Affiliations:** 1 Anadolu University, Faculty of Education, 26470, Eskişehir, Turkey; 2 Anadolu University, Faculty of Pharmacy, Department of Biochemistry Science, 26470, Eskişehir, Turkey

**Keywords:** Beta-catenin, Epithelial ovarian cancer, Fibronectin, GSK-3beta, Integrin

## Abstract

**Objective(s):**

This study examines the impact of integrin-linked kinase (ILK), protein kinase B (AKT), glycogen synthase kinase-3β (GSK-3β), and β-catenin signal molecules in SKOV-3 ovarian cancer cells adhered to fibronectin.

**Materials and Methods:**

Expression levels of α4, αv, β1, and β6 integrin subunits known as the fibronectin ligand were investigated with the flow cytometry technique. The effects of ILK, AKT, GSK-3β, and β-catenin on the binding of SKOV-3 cells to fibronectin were examined by using the Real-Time Cellular Analysis (RTCA) method. Additionally, the interaction of these proteins was investigated by using Western blot analysis.

**Results:**

The results show that the expression levels of integrin subunits were ranked as αv (67.8%), followed by α4 (48.55%), β6 (32.05%), and β1 (31%) on SKOV-3 cells. RTCA results showed that ILK (10 µM Cpd22), GSK-3β (50 μM GSK-3β inhibitor-XI), AKT (35 µM FPA 124), and β-catenin (50 μM cardamonin) inhibitors decreased significantly (*P*<0.01) binding to fibronectin at 24 hr. Western studies in SKOV-3 cells adhered to fibronectin have shown that in inhibition of ILK, AKT expression was strongly inhibited, whereas, in the inhibition of AKT, ILK expression was strongly inhibited. Furthermore, the expression of β-catenin is partially reduced in inhibition of these two molecules. In β-catenin inhibition, AKT and ILK expressions are also strongly inhibited.

**Conclusion:**

ILK, AKT, GSK-3β, and β-catenin were found to be fundamental molecules in binding of SKOV-3 cells to fibronectin. ILK and AKT affect strongly the level of expression of each other, and both also affect the signal path of β-catenin.

## Introduction

Ovarian cancer is the seventh most diagnosed cancer among women worldwide and a cancer type with a high mortality rate compared with other cancer types (1). One of the reasons for high mortality is associated with the diagnosis of the disease at an advanced stage (2). Another reason is the resistance of ovarian cancer to the combination of platinum-based therapies such as cisplatin, carboplatin, and paclitaxel. Chemotherapy resistance is a serious problem in the treatment of ovarian cancer (3). It is thereby quite important to elucidate the effect of binding and signaling pathways, especially in metastatic ovarian cancer.

In many human carcinoma cases, metastasis occurs when the tumor cells gradually migrate to the distant sites through blood vessels. Metastasis with blood vessels, on the other hand, is rare in ovarian carcinoma. Metastasis occurs mostly by direct extension from the ovarian/fallopian tumor to adjacent organs, or by separation of cancer cells from the primary tumor. The cells separated from the tumor carried by the peritoneal fluid bind to the abdominal peritoneum or omentum. The first stages of metastasis are regulated by a controlled interaction of adhesion receptors and proteases (4). 

Fibronectin, an extracellular matrix (ECM) protein, has been reported to be overexpressed in the metastasis stage of ovarian cancer (5). Integrins, which are cell adhesion receptors, bind directly to ECM components such as fibronectin. Integrins have an important role in the survival of tumor cells and can affect tumor cell survival positively or negatively, with or without ligand binding. Integrin receptors are composed of combination of 18 α and 8 β subunits (6). β1, β6, αv, and α6 of the integrin subunits can be able to bind to fibronectin (7). 

It is known that the first receptors of the signal transmitted after the binding of the integrins with the ECM components are Integrin-linked Kinases (ILK) (8). ILK is a serine/threonine protein kinase that interacts with the cytoplasmic β1 and β3 subunits of integrins. ILK regulates signals from integrin-ECM interactions and growth factors. ILK is involved in many cellular processes such as cell survival, migration, and invasion at the onset of the interaction between integrin and ECM (9, 10). 

It was reported that ILK is overexpressed in many cancers, such as colon (11), prostate (12), and ovarian cancers (13). Overexpression of ILK is associated with tumor growth, invasion, and metastatic tumors (13, 14). The expression of ILK also has been increased with ovarian tumor grade. As the dissolving factors in the peritoneal tumor fluid mediate continuous overexpression of ILK, ILK expression increases in the progression of ovarian cancer (13). It has been also found that ILK regulates the metastatic behavior of ovarian cancer cells (15). 

ILK is known to be an important molecule in the regulation of many signaling pathways. ILK affects AKT, GSK-3β, and β-catenin signaling molecules (16). Active AKT plays a role in signaling pathway metabolism, growth, proliferation, and survival (17). GSK-3β signal pathway has a role in proliferation, cell survival, cell death (16), and invasion. In addition, GSK-β3 affects many signal pathways including Wnt/β-catenin, which has a role in differentiation, apoptosis, migration, and epithelial-mesenchymal transition (18).

Damage to the cellular signal pathway plays an important role in the proliferation, survival, invasiveness, and metastasis of tumor cells. Identification of integrin-induced ovarian cancer cell signaling pathways will shed light on the new treatment modalities in the metastatic stage where mortality is high. This study focused on investigation of the effects of ILK on β-catenin, GSK-3β, and AKT signaling molecules after SKOV-3 ovarian cells bound to fibronectin. Firstly, expression of integrins was measured on SKOV-3 cells bound to fibronectin. Secondly, the role of ILK, β-catenin, GSK-3β, and AKT in cell binding and the expression of ILK, β-catenin, GSK-3β, and AKT were investigated using cell-binding assay and Western blot assay, respectively. Specific inhibitors against the molecules mentioned above were used to investigate the interaction of molecules with each other. 

## Materials and Methods

***Cell culture***

Human ovarian cancer (SKOV-3, ATCC® HTB-77™) cells were obtained from ATCC. SKOV-3 cells were cultured in DMEM, 10% Foetal Bovine Serum (Gibco, UK), 0.1 mM MEM Non-Essential Amino Acids (Sigma-Aldrich, UK), 2 mM L-glutamine, and 1% penicillin-streptomycin. The cells were subcultured using 0.25 % trypsin-EDTA solution (Sigma-Aldrich, UK) every two days. Exponentially growing cultures were maintained in an incubator with a humidified atmosphere with 5 % CO_2_/95 % air at 37 °C.

***Measurement of integrin expressions***

Expression levels of β1, β6, αv, and α4 integrin subunits on SKOV-3 cells were quantified by flow cytometry. For the analysis, 5 x 10^5^ cells/ml were collected using 1 × trypsin, washed twice with 1 ×PBS, and added to each microwell. The cells were first treated with primary antibodies, anti-integrin α4 (sc-14008), αv (sc-376156), β1 (sc-374429), and β6 (sc-15329) for 30 min at 4 °C and then FITC conjugated secondary antibody (sc-2010) for 30 min at 4 °C. For negative control, the first antibody or FITC secondary antibody was omitted. All samples were washed twice with 1 ×PBS and analyzed using a flow cytometer (Becton Dickinson, FACSAria II, Canada). 

All experiments were performed twice independently. The net ratios of the integrin subunit’s expression were calculated by subtracting the control value (integrin subunit’s P3-Control P3) and then the averages of both test results were calculated.

***Investigation of the role of the signal molecules in the binding ability of SKOV-3 cells to fibronectin***

The role of ILK, AKT, GSK-3β, and β-catenin in the binding ability of SKOV-3 cells to fibronectin or poly-l-lysine was investigated using a real-time cellular analysis system (RTCA; xCELLigence, Roche) and RTCA software version 1.1. The system is used in gold electrodes at the bottom of the plates and measures the changes in electrical impedance. The electrical impedance is expressed as cell index (CI) value which calculates the number of the cells attached and spread on the bottom of the well. CI was calculated as follows:

CI = (resistance measured at a time point ‒ resistance measured without the cell) / 15Ω

First, 16-well plates were incubated with 50 μg/ml fibronectin, and 10 μg/ml poly-l-lysine or uncoated for control for 1 hr at 37 °C. After that, the plates were washed with 1% TBS twice and incubated with 1 % BSA for 1 hr at room temperature. 5 × 10^4^ SKOV-3 cells/ml were seeded into plates and placed into RTCA for 2 hr incubation. The cells were treated with different concentrations of either ILK inhibitor (Cpd 22) (Calbiochem, 407331; 5-10 µM), AKT inhibitor (FPA 124) (Santa Cruz, CAS 902779-59-3; 35-50 μM), GSK3-β inhibitor (Calbiochem, 361553; 25-50 μM) or β-catenin inhibitor (Cardamonin) (CAS 19309-14-9; 10-100 μM). The impedance was monitored at 15-min intervals for a period of 48 hr. No inhibitor was added to all control groups. Poly-l-lysine was used as a control binding protein. The cells were able to bind to poly-l-lysine but not spread. In every experiment, each concentration was applied in triplicate and each experiment was repeated twice independently of each other.

***Expression levels of the signal molecule proteins after the cells bound to fibronectin***

The plates were coated with 50 µg/ml fibronectin or 10 μg/ml poly-l-lysine. 5 × 10^6^ cells/ml were seeded into the Petri dish and incubated for 1 hr at 37 °C. The cells were treated with 10 µM of ILK inhibitor (Cpd22) for 12 hr, 50 µM of GSK-3β inhibitor XI for 72 hr, 50 µM of β-catenin inhibitor (cardamonin) for 72 hr, and 35 µM of AKT inhibitor (FPA 124) for 24 hr at 37 °C. The concentrations of the inhibitors were applied according to the concentrations determined by the binding assay. Incubation times were determined by the time the inhibitor affected protein expression. After twice washing with cold PBS, the cells were lysed in lysis buffer (50 mM Tris-HCl, pH 7.9, 10 mM NaCl, %1 Nonidet P-40, 10 mM Na_3_VO_4_, 40 mM NaF, 1 mM DTT, 1 mM PMSF, 1 µg/ml aprotinin and leupeptin) on ice for 20 min. The cells were scrapped with a cell scraper and then the cell lysates were transferred into Eppendorf tubes. The cells were centrifuged at 13 000 rpm at 4 °C. The supernatant was then transferred into a clean cold tube. Total protein concentration was detected by using Bradford dye in a spectrophotometer. Equal amounts of protein samples were loaded onto 10% acrylamide gels. Afterward, the gel was then transferred to a polyvinylidene (PVDF) membrane in a blotting apparatus for 2 hr at 40 mA, blocked with 5% bovine serum albumin for 1 hr at room temperature, and washed in TBS with 0.1 Tween-20. The membranes were then treated with primary antibodies anti-AKT (sc-5298), anti-ILK (sc-137221), anti-β-catenin (sc-7963), anti-GSK-3β (sc-81462), anti-phospho-AKT-1 (sc-33437), and anti-actin (sc-7210) for 1 hr at room temperature. After that, the membranes were incubated with anti-mouse IgG or anti-goat IgG conjugated with horseradish peroxidase (HRP), a secondary antibody, for 1 hr at room temperature. After the membranes were washed with Tween containing PBS, they were incubated with 3,3′,5,5′-Tetramethylbenzidine (TMB) (T0565, Sigma-Aldrich) at room temperature until images appeared. Finally, the protein bands were scanned by using UVP (Bio spectrum 510 Imaging System) (19).

***Statistical analysis***

The results of two independent RTCA experiments (n=6) were analyzed using the t-test to check if the binding of control and experiment groups are statistically different (**P≤0.01; *P≤0.05).

## Results

***Expression levels of integrins on SKOV-3 ovarian cancer cells***

**Figure 1:**
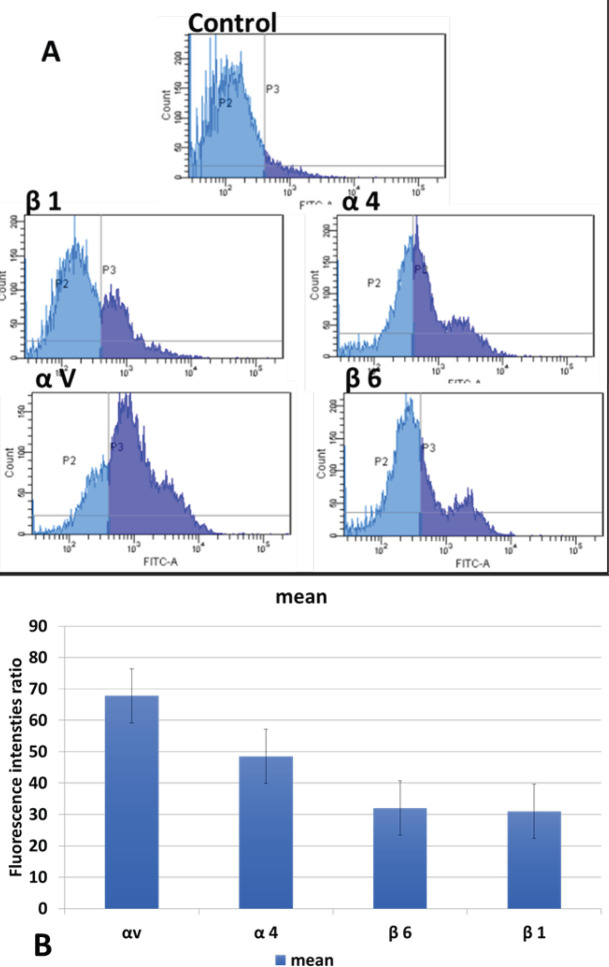
Integrin subunit expression staining with specific integrin mAbs. One of the two independent experiments is shown on the graph (A). Mean of the net expression levels of the two independent experiments (B)

Expression levels of αv, α4, β1, and β4 integrins determined by flow cytometry are shown in Figure 1. Labeling of SKOV-3 cells with anti-αv mAb showed that these cells expressed a high level of αv on their surface compared with the other integrins that were investigated. The percentage of expression levels of integrin subunits were highest at αv (67, 8%), then α4 (48, 55%), β6 (32, 05%), and β1 (31%) according to the mean of two independent experiments (Figure 1). 

***Effects of the signal molecules on the binding of SKOV-3 cells to fibronectin***

The effects of ILK, AKT, GSK-3β, and β-catenin on the binding of SKOV-3 cells to fibronectin were analyzed using the RTCA system in the presence and absence of the inhibitors.

*The effects of ILK on SKOV-3 cells adhered to fibronectin*

**Figure 2:**
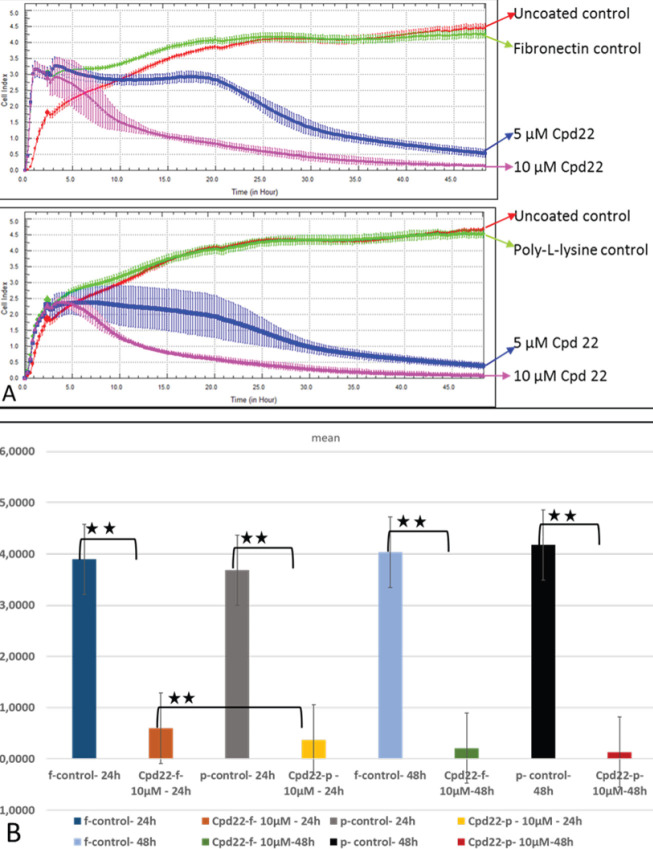
Binding rates of SKOV-3 cells to fibronectin or poly-l-lysine in the presence of various concentrations of Cpd22 inhibitor and the statistical analysis of the results. The graph was plotted with the mean of three independent wells for each concentration. One of the two independent experiments is shown in (A). The differences of binding of SKOV-3 cells to either fibronectin or poly-l-lysine after treatment with 10 mM Cpd22 for 24 or 48 hr were examined by the t-test (B). The figures presented are the means of two independent experiments (n=6). In each experiment, the average of three wells was used (**P≤0.01, *, *P*≤0.05)

The various concentrations of a specific ILK inhibitor were added into each well coated with either fibronectin or poly-l-lysine and monitored at 48 hr (Figure 2A). After inhibition of ILK, the binding rate of SKOV-3 cells to fibronectin and poly-l-lysine was declined at different levels depending on both inhibitor concentrations and time periods. The lower concentration (5 µM of Cpd22) of inhibitor affected the binding of cells to both fibronectin and poly-l-lysine in the later time points (its effects exist about after 20 hr of incubation time) compared with higher concentration (10 µM of Cpd22) of inhibitor effects. 10 µM Cpd22 was determined as an effective dose and statistical analyses were performed.

The results showed that 10 µM Cpd22 reduced the binding of SKOV-3 cells to fibronectin and poly-l-lysine compared with the control group both at 24 and 48 hr. The reduction was statistically significant at 1% at 24 hr and 48 hr (Figure 2B). The inhibition of ILK further reduced the binding of cells on poly-l-lysine rather than fibronectin when Cpd22 is applied to the cells for 24 h. However, after 24 hr incubation time, the inhibition of ILK reduced both binding and spreading of cells on poly-l-lysine and fibronectin, respectively (*P*<0.01). The results obtained indicated that ILK is an essential component for both binding and spreading of SKOV-3 cells as predicted.

*Effects of GSK-3β on SKOV-3 cells adhered to fibronectin*

**Figure 3:**
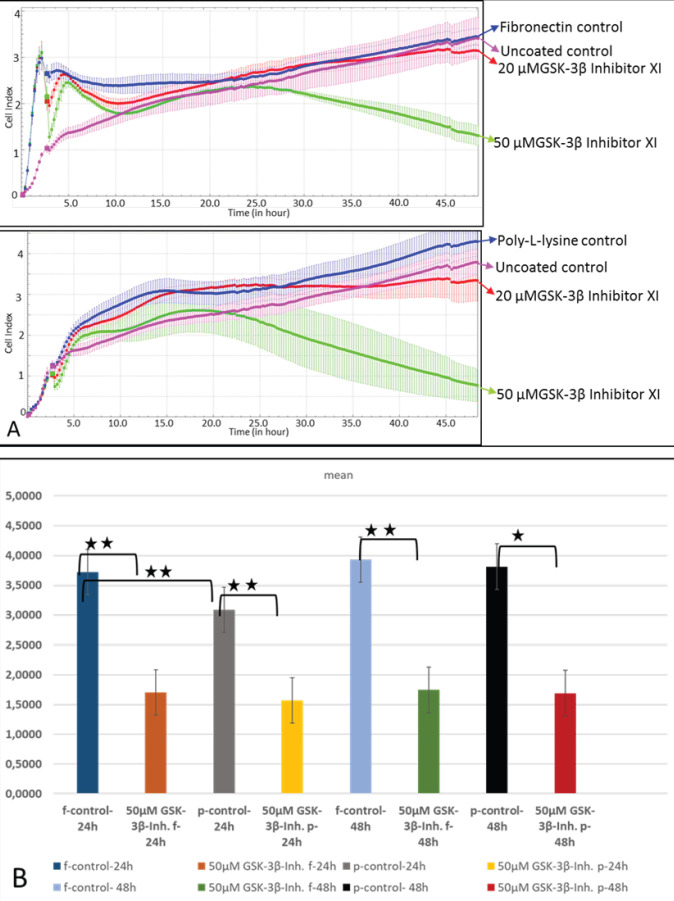
Binding rates of SKOV-3 cells to fibronectin or poly-l-lysine in the presence of either 20 or 50 µM of GSK-3β inhibitor XI and statistical analysis of the results. The graph was plotted with the means of three wells for each concentration. One of the two independent experiments is shown in (A). The differences in binding levels of SKOV-3 cells treated with 50 μM GSK-3β inhibitors XI to poly-l-lysine/fibronectin at 24 and 48 hr were tested by t-test (B). The data presented are the means of two independent experiments (n=6). In each experiment, it contains the average of three wells (** *P*≤0.01, * *P*≤0.05)

The cells treated with 20 and 50 µM of GSK-3β inhibitor XI were used to investigate the effects of GSK-3β molecules on the binding of SKOV-3 cells to fibronectin or poly-l-lysine (Figure 3A). The results showed that 50 µM of GSK-3β inhibitor XI began to decrease the binding of SKOV-3 cells more dramatically than 20 µM of GSK-3b inhibitor after 25 hr (Figure 3A). For this reason, the dose of 50 μM GSK-3β inhibitor XI was determined as the effective dose for the reduction of binding. 50 μM GSK-3β inhibitor XI significantly reduced the binding of SKOV-3 cells to fibronectin and poly-l-lysine compared with the control group at 24 and 48 hr (Figure 3B). Poly-l-lysine and fibronectin groups were compared to determine the effect of fibronectin on binding. There was a statistically significant difference between the poly-l-lysine and fibronectin control groups at 24 hr, but this difference was not indicated after 48 hr incubation. 

*Effects of β-catenin on SKOV-3 cells adhered to fibronectin*

**Figure 4:**
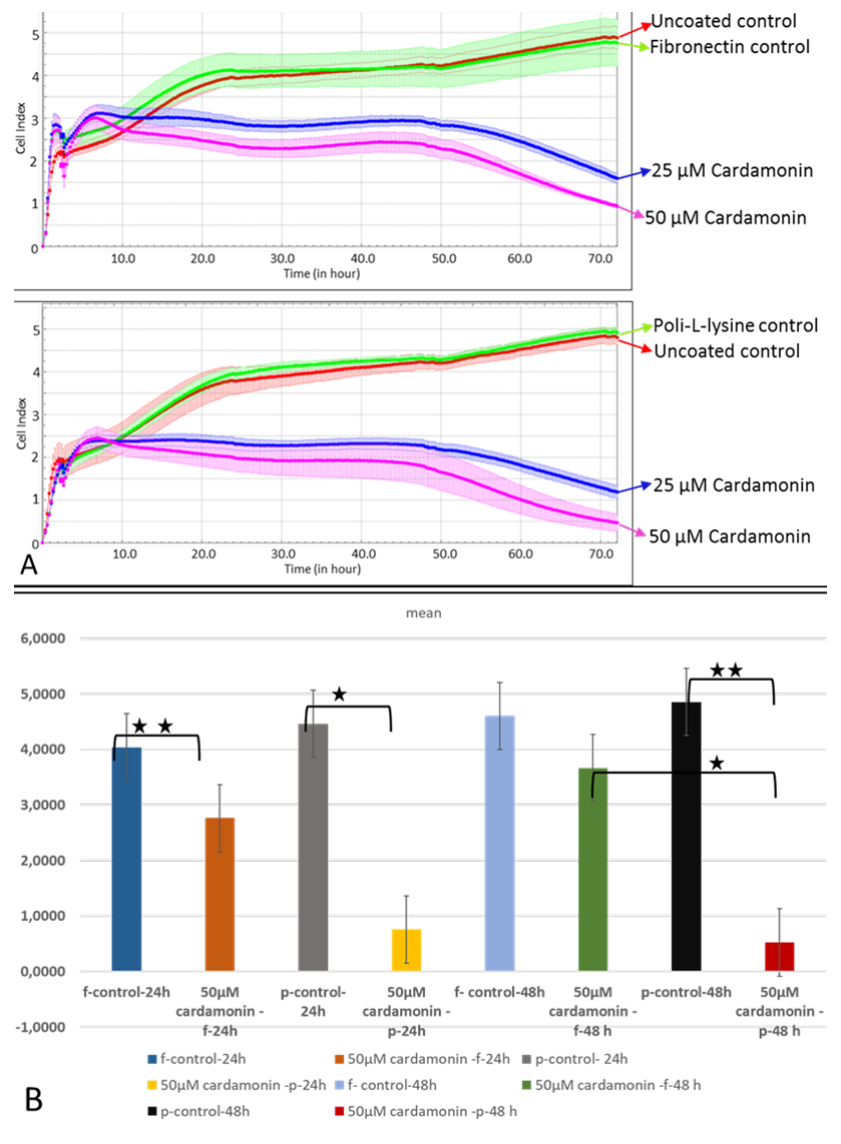
Binding rates of SKOV-3 cells to fibronectin or poly-l-lysine in the presence of 25 or 50 µM concentrations of cardamonin and statistical analysis of the results. The graph was plotted with the mean of three wells for each concentration. One of the two independent experiments is shown (A). The differences in the binding of SKOV-3 cells treated with 50 μM cardamonin to poly-l-lysine/ fibronectin after 24 and 48 hr were tested by t-test (B). The data presented are the means of two independent experiments (n=6). In each experiment, it contains the average of three wells (**P≤0.01, * *P*≤0.05)

SKOV-3 cells were incubated with different concentrations of cardamonin, Wnt/ β-catenin inhibitor, to determine the effect of β-catenin on the binding of cells to fibronectin or poly-l-lysine. It was found that cardamonin inhibited the binding of SKOV-3 cells to fibronectin and poly-l-lysine in a dose-dependent manner (Figure 4A). The inhibition of β-catenin with 50 μM cardamonin decreased the binding of SKOV-3 cells to fibronectin and poly-l-lysine compared with the control group after 24 incubation time, significantly (Figure 4B). It was also observed that after SKOV-3 cells were treated with 50 μM cardamonin for 48 h, the cell-binding significantly decreased in the poly-l-lysine group compared with the fibronectin group (*P*<0.05). This shows that β -catenin plays a significant role during the assembly of the adhesion complex of SKOV-3 cells. 

*Effects of AKT on SKOV-3 cells adhered to fibronectin*

**Figure 5:**
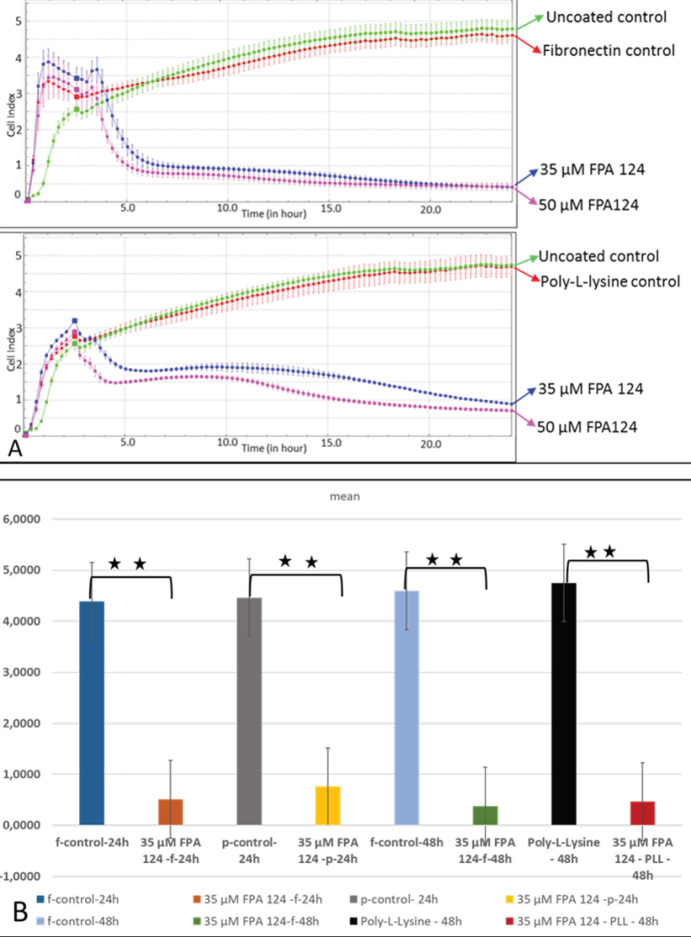
Binding rates of SKOV-3 cells to fibronectin or poly-l-lysine in the presence of different concentrations FPA 124 inhibitor and statistical analysis of the results. The graph was plotted with the mean values of three wells for each concentration. One of the two independent experiments is shown (A). The differences in binding of SKOV-3 cells treated with 35 μM FPA 124 inhibitor to poly-l-lysine/ fibronectin at 24 and 48 hr were tested by t-test (B). The data presented are the means of two independent experiments (n=6). In each experiment, it contains the average of three wells (** *P*≤0.01, * *P*≤0.05)

The binding rate of SKOV-3 cells to fibronectin or poly-l-lysine was measured at different concentrations of FPA124, a specific inhibitor of AKT. The results show that 35 and 50 µM concentrations of FPA 124 tested were able to decrease the binding of SKOV-3 cells to fibronectin and poly-l-lysine at the same level after 48 hr incubation (Figure 5A). After treatment with 35 μM of FPA, the binding level of SKOV-3 cells to fibronectin or poly-l-lysine was significantly decreased as compared with the control group at 24 hr and 48 hr (*P*<0.01). (Figure 5B). Comparison of 35 μM of FPA 124 treated cells adhered on fibronectin or poly-l-lysine showed that the binding decreased on fibronectin group compared with the poly-l-lysine group after 24 hr incubation time point. But the reduction was not statistically significant. 

As a result, the inhibition of both AKT and ILK decreased the effective binding in a short time. According to these results, ILK and AKT signaling molecules firstly might be involved in the binding process of SKOV-3 cells to fibronectin and then the other proteins are activated somehow.

***Protein expression***

*Effects of ILK on the signal molecules*

**Figure 6:**
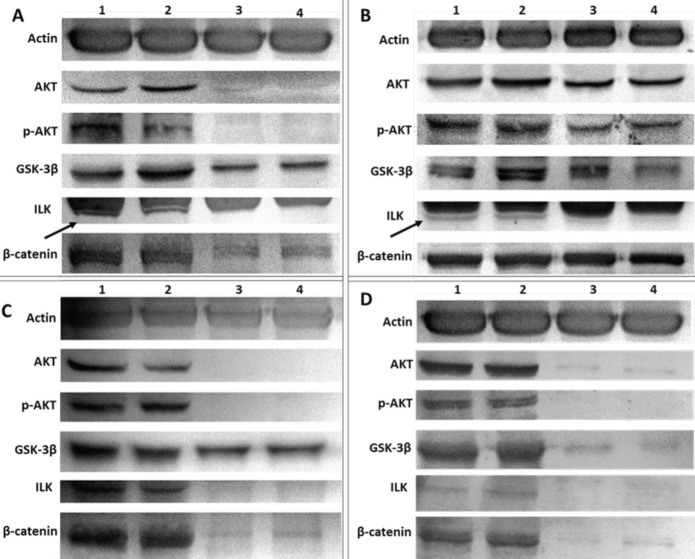
Protein expression after treatment of SKOV-3 cells with various inhibitors attached to fibronectin or poly-l-lysine. The expression levels of ILK, Akt, p-AKT, GSK-3β, and β-catenin proteins after treatment of SKOV-3 cells with 10 μM CpD22 inhibitor (A), with 50 µM GSK-3β inhibitor XI (B), with 50 μM cardamonin inhibitor (C), with 35 µM FPA 124 inhibitor (D). 1-Fibronectin, 2-Poly-l-lysine, 3-Fibronectin+the inhibitor, 4-Poly-l-lysine+the inhibitor were tested

The effects of ILK on the expression of the signaling molecules were examined after SKOV-3 cells adhered to fibronectin or poly-l-lysine in the presence or absence of ILK inhibitor, Cpd22 (Figure 6A). The expression of the ILK protein was completely inhibited by Cpd22 after the cells adhered to both fibronectin and poly-l-lysine. These data indicated that ILK protein expression inhibited cell spreading and adhesion. The results obtained here also showed that the expression of GSK-3β, AKT, and β-catenin proteins were decreased in both conditions whereas p-AKT expression was completely inhibited by inhibition of ILK protein expression. Shortly, the expression ILK protein directly regulates the expression of p-AKT and AKT proteins. ILK protein probably causes the activation of p-AKT during the adherence and spread of SKOV-3 cells. ILK also leads to partial activation of GSK-3β and β-catenin proteins.

*Effects of GSK-3β on the signal molecules*

The effects of GSK-3β protein on AKT, ILK, and β-catenin protein expression were investigated in SKOV-3 cells in the presence or absence of GSK-3β inhibitor XI (Figure 6B). In the presence of 50 μM GSK-3β inhibitor XI, there was no change in GSK-3β protein expression in the fibronectin group, but the GSK-3β protein expression decreased significantly in the poly-l-lysine group. On the other hand, the expression of ILK, p-AKT, and AKT decreased in the fibronectin group in the presence of 50 μM GSK-3β inhibitor. The expression levels of GSK-3β, ILK, p-AKT, AKT, and β-catenin proteins decreased in SKOV-3 cells adhered to poly-l-lysine in the presence of 50 μM GSK-3β inhibitor XI. As a result, the inhibition of GSK-3β protein expression slightly decreased the expressions of p-AKT, AKT, and ILK in SKOV-3 cells adhered to either fibronectin or poly-l-lysine that indicated in these circumstances, GSK-3β protein might be related to all these proteins indirectly. The results in our experimental conditions showed that GSK-3β inhibitor XI reduced GSK-3β protein expression significantly in the poly-l-lysine group, where the cells were bound but not spread, whereas the inhibitor did not affect GSK-3β protein expression in the fibronectin group where the cells were bound and spread. In Figure 6A, it was shown that ILK leads to partial activation of GSK-3β affecting the ILK signaling pathway partially (Figure 6B). 

*Effects of β-catenin on the signal molecules*

The impacts of β-catenin protein on AKT, GSK-3β, and ILK protein expressions were examined in SKOV-3 cells (Figure 6C). Cardamonin inhibited β-catenin expression significantly in both fibronectin and poly-l-lysine groups. It was also found that the GSK-3β expressions decreased, while AKT, p-AKT, and ILK expressions were almost inhibited after incubation with cardamonin in SKOV-3 cells adhered to fibronectin or poly-l-lysine (Figure 6C). Importantly, inhibition of β-catenin protein expression by cardamonin has been shown to directly affect the expression of the ILK protein. In addition, inhibition of the β-catenin signaling pathway influenced AKT and p-AKT, while GSK-3β was affected partially (Figure 6C). 

*Effects of AKT on the signal molecules*

The effects of AKT protein on GSK-3β, ILK, and β-catenin protein expressions were shown in the presence or absence of FPA124 after SKOV-3 cells adhered to fibronectin or poly-l-lysine (Figure 6D). The results showed that AKT, p-AKT, and ILK expressions were significantly inhibited, while GSK-3β and β-catenin protein expressions decreased on both fibronectin and poly-l-lysine in the presence of FPA124 inhibitor (Figure 6D). The results indicated that inhibition of AKT expression directly affected ILK expression as shown in Figure 6C. As predicted, inhibition of AKT protein also directly inhibited p-AKT expression. Finally, the AKT signaling pathway has been shown to partially influence GSK-3β and β-catenin signal pathways.

**Figure 7:**
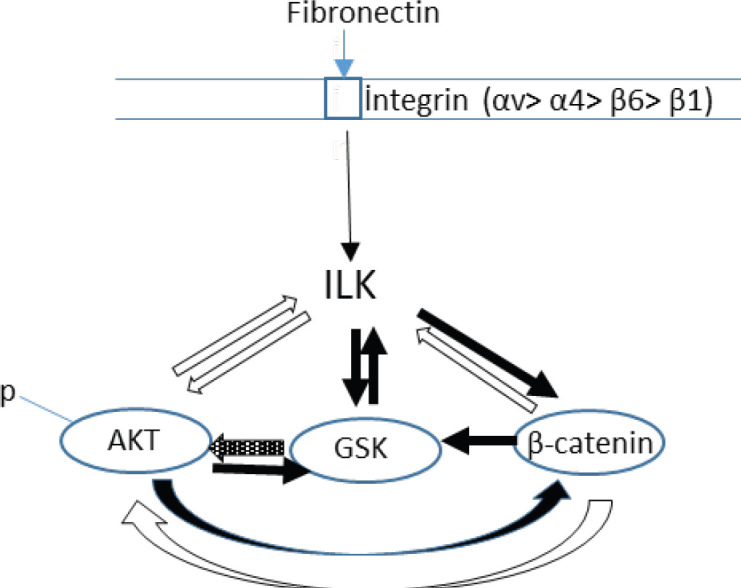
Interactions of ILK, GSK-3β, AKT, p-Akt, and β-catenin proteins after engagement of integrins with fibronectin on SKOV-3 cells. : strong interaction; partial interaction; weak interaction. The figure has been drawn according to the data in Figure 6

## Discussion

Outside-in and inside-out signaling are a hallmark of the processing of cancer cells as well as ovarian tumor cells. In this study, the interactions of ILK, p-AKT, GSK-3β, and β-catenin proteins in SKOV-3 cells adhered to extracellular protein, fibronectin via integrin receptors were investigated. 

Fibronectin, overexpressed in ovarian cancer patients undergoing omental metastasis, plays an important role in invasion, and especially metastasis of ovarian cancer cells. (5). Integrins play a role in cell survival and growth after interaction with ECM and that affects many signal pathways (20). It is known that the binding capacities of integrins in cancer cells are suppressed or stimulated (21). In this study, the expressions of α4, αv, β1, β6 integrin subunits were shown on SKOV-3 cells that interact with fibronectin. It was found that the expression level of αv integrin subunit was higher than other integrin subunits on SKOV-3 cells. The expression levels of integrin subunits were mostly arranged at αv, then α4, β6, and β1 on SKOV-3 cells. High levels of αv expression were associated with metastasis (20, 22).

ILK is the first molecule of the signal transmitted after interaction of integrin-ECM and interacts with the cytoplasmic β1 and β3 subunits of integrins (8). Cruet-Hennequart *et al*. (2003) informed that αv integrin subunits regulate cell proliferation via ILK in ovarian cancer (23). Lössner *et al*. (2009) also showed that ILK is up-regulated due to αvβ3 integrin in ovarian cancer (24). In this studey, we demonstrated the expression of αv and β1 integrin subunits, which is known to be effective on the ILK signaling pathway, in ovarian cancer cells, SKOV-3. ILK expression is also known to be high in advanced ovarian tumors (13). A study (2004) reported that immunoreactive (ir) ILK is an ovarian tumor antigen and that the expression of irILK in the serum of patients with grade 1,2,3 ovarian cancers is 6–9 times greater than the serum of normal patients with benign tumors (25).

In recent years, various studies have been carried out on the silencing and inhibition of the ILK gene in ovarian cancer. Silencing of the ILK gene induces apoptosis in SKOV-3 cells (26), blocks tumor growth in nude mouse xenografts (27). Bruney *et al*. (2016) reported that down-regulation of ILK expression or activity reduces the adhesion and invasion of ovarian cells to collagen gels (15). Our results showed that inhibition of ILK signal molecules by the Cpd22 inhibitor decreased the binding of SKOV-3 cells to fibronectin significantly. We showed that β-catenin, GSK-3β, and AKT, and especially ILK are important molecules in the binding of fibronectin to ovarian cancer cells. 

Some other studies are showing that these molecules (β-catenin, GSK-3β, AKT, and ILK) are involved in normal biological events, and their overexpression is associated with cancer. A study (2002), for instance, reported that while AKT expression was observed in colorectal cancer, it was not detected in the normal colon mucosa. They also found that overexpression of AKT is associated with colon carcinogenesis (28). It was reported that ILK is required for the assembly of matrix-forming adhesions in endothelial cells (29). The association of ILK with overexpression in many cancers has been reported (11-13). The β-catenin protein was first discovered as part of the adherent linkage complex (30). However, its inappropriate activation in other tissues has often been reported to cause cancer (31). Rask *et al*. (2003) reported that β-catenin and GSK-3β were expressed in ovarian cancer cells (OVCAR-3) and immortalized human ovarian surface epithelial cells (IOSE) as the control. However, β-catenin and GSK-3β nuclear staining were found only in OVCAR-3 cells, not in IOSE. They also demonstrated that β-catenin and GSK-3β expressions were increased in ovarian adenocarcinomas compared with the normal ovary (32).

AKT, serine/threonine kinase, is a pathway that can be activated by various signaling pathways such as integrins and growth factors. Researchers (2008) disclosed that β1 integrins activate the PI3K/AKT pathway by phosphorylation of AKT at Ser473 and Thr308 during cell adhesion and spreading. It has also been disclosed that this activity occurs independently of EGF receptor activity and focal adhesion kinase (33). Similarly, it was reported here that the expression of β1 integrin subunit was detected on SKOV-3 cells and that the AKT signaling pathway influenced the binding to fibronectin of SKOV-3 cells. In addition, the expressions of AKT and p-AKT were observed in SKOV-3 cells adhered to fibronectin.

ILK may play a role in the regulation of many signaling pathways after integrin interaction. After ILK interacts with the integrins, ILK phosphorylates ser473 residues of AKT molecule to activate the PKB/AKT signaling pathway that is responsible for cell growth and proliferation. It has been shown that kinase deficient of ILK inhibited phosphorylation of PKB/AKT *in vivo* (16). Likewise, in our study, ILK and AKT signal pathways strongly influence each other after integrin fibronectin interaction in SKOV-3 cells (Figure 7). 

ILK also affects the GSK-3β signal pathway; serine/threonine protein kinase phosphorylates GSK-3β to inhibit GSK-3β activity (16). GSK-3β is especially responsible for tumor invasion and treatment resistance. The impact of GSK-3β on cancer however is controversial. Depending on cell type and phosphorylation status, GSK-3β may show tumor suppressor or promoter effect (18). Our results showed that inhibition of ILK could not completely inhibit GSK-3β expression. ILK and GSK-3β are signal pathways that partially affect each other in SKOV-3 cells (Figure 7). It is known that GSK-3β is also regulated by many signal pathways including Wnt/β-catenin and PI3K-AKT-mTOR, which control cell growth and proliferation (18). The results obtained here showed that when the Wnt/β-catenin pathway was inhibited using cardamonin on fibronectin-bound SKOV-3 cells, a significant reduction in the expression of the GSK-3β molecule was observed. This result showed the importance of the Wnt/β-catenin pathway in the GSK-3β signaling pathway. As a result, we showed that the signal path of β-catenin partially affected the signal path of GSK-3β. In addition, while the AKT signaling path partially affected both GSK-3β and β-catenin molecules, the GSK-3β signaling pathway weakly affected the AKT signaling pathway (Figure 7).

β-catenin, an important molecule of the Wnt signaling pathway, acts as a cell-cell binding in the cell membrane and as a transcriptional regulator of specific genes in the nucleus (34). Cardamonin was used to investigate the effect of β-catenin on the other signaling molecules and adhesion in our study. It has been reported that cardamonin blocked the Wnt/β-catenin signaling pathway through degradation of β-catenin (35). In recent years, the effect of cardamonin on cancer and signaling pathways has been investigated. It was announced that cardamonin had an anti-inflammatory effect by suppressing mTOR in ovarian cancer (36). In our previous study, we demonstrated that cardamonin reduced cell proliferation and induced apoptosis on ovarian cancer cells via down-regulating NF-κB and cyclin D1 (37). In this study, it was observed that ILK, AKT, and p-AKT signal molecule expressions were strongly affected, while GSK-3β was also partially affected when the β-catenin molecule was inhibited by cardamonin. In addition, it was found that ILK and AKT signaling pathways were partially effective on β-catenin signaling molecule expression in SKOV-3 cells adhered to fibronectin (Figure 7). ILK and AKT expressions were decreased by both GSK-3β and β-catenin inhibitors. However, our study found that β-catenin expression did not change on SKOV-3 cells adhered to fibronectin after inhibiting the GSK-3β pathway using GSK-3β inhibitor XI, but slightly decreased on SKOV-3 cells adhered to poly-l-lysine. Since GSK-3β inhibitor XI could not inhibit GSK-3β expression on fibronectin bound to the cells, no direct effect of β-catenin on the molecule could be observed. However, when the Wnt/β-catenin pathway was inhibited by cardamonin, both GSK-3β and β-catenin expression decreased importantly, implying that the Wnt signaling pathway was highly effective on both GSK-3β and β-catenin.

Our study also indicated that β-catenin plays a role in the adhesion of SKOV-3 cells. We showed that inhibition of β-catenin molecules by cardamonin decreases binding of SKOV-3 cells to fibronectin significantly (*P*≤0.01) at 24 hr. It was also demonstrated that ILK, AKT, and GSK-3β are very important molecules in the binding of SKOV-3 cells to fibronectin. Furthermore, it was shown that ILK and AKT strongly affect each other’s expressions and that these two molecules also partially affect the β-catenin signal molecule necessary for cell binding. According to our results, the effect of these two molecules on binding is probably realized via β-catenin. Like our results, it has been reported that ILK is a key molecule in the progression of human colon cancer, possibly by *in vivo* regulation of β-catenin, E-cadherin, and AKT pathways (11). 

## Conclusion

This study demonstrated that ILK, AKT, GSK-3β, and β-catenin are important molecules in binding of SKOV-3 cells to fibronectin. Additionally, it was found that ILK and AKT signaling molecules reduce binding in a shorter time and more effectively. Moreover, these two signal paths, ILK, and AKT, affect each other very strongly and both also affect the signal path of β-catenin, which is an important element in binding. The inhibition of β-catenin by cardamonin also strongly influences ILK and AKT signaling pathways. Another finding of fibronectin-dependent SKOV-3 cells is that the GSK-3β signaling pathway is affected by both ILK and Wnt/β-catenin signaling pathways. 

## Authors’ Contributions

SMSK, Zİ Study conception and design; SMSK, Zİ Data analsis and draft manuscript preparation; SMSK Critical revision of the paper; SMS K, Zİ Supervision of the research; SMSK, Zİ Final approval of the version to be published.

## Conflicts of Interest

No potential conflicts of interest/competing interests were reported by the authors.
